# Protective Effects of Repetitive Transcranial Magnetic Stimulation Against Streptozotocin-Induced Alzheimer’s Disease

**DOI:** 10.1007/s12035-023-03573-8

**Published:** 2023-09-27

**Authors:** Seul-Ki Kim, Geun Yong Lee, Su Kang Kim, Yong-Jin Kwon, Eun-Bi Seo, Haeri Lee, Song-Hee Lee, Sung Joon Kim, Sangsik Lee, Sang-Kyu Ye

**Affiliations:** 1 Department of Pharmacology and Biomedical Sciences, Seoul National University College of Medicine, Seoul, 03080 Republic of Korea; 2 Remed Co. Ltd., 21-7, Weeleseoilo 1, Seongnam, Korea; 3 Department of Biomedical Laboratory Science, Catholic Kwandong University, Gangneung, Korea; 4 Ischemic/Hypoxic Disease Institute, Seoul National University College of Medicine, Seoul, 03080 Republic of Korea; 5 Department of Cosmetic Science, Kyungsung University, Busan, 48434 Republic of Korea; 6 Biomedical Science Project (BK21PLUS), Seoul National University College of Medicine, Seoul, 03080 Republic of Korea; 7 Department of Physiology, Seoul National University College of Medicine, Seoul, 03080 Republic of Korea; 8 Department of Biomedical Engineering, Catholic Kwandong University College of Medical Convergence, Gangneung, 25601 Republic of Korea; 9 Wide River Institute of Immunology, Seoul National University, Hongcheon, 25159 Republic of Korea; 10 Neuro-Immune Information Storage Network Research Center, Seoul National University College of Medicine, Seoul, 03080 Republic of Korea

**Keywords:** Repetitive transcranial magnetic stimulation (rTMS), Phosphorylation of intracellular signaling pathways, Alzheimer’s disease, Cognitive impairment improvement, Neuroprotective effects

## Abstract

Repetitive transcranial magnetic stimulation (rTMS) is a non-invasive brain stimulation under investigation for treatment of a wide range of neurological disorders. In particular, the therapeutic application of rTMS for neurodegenerative diseases such as Alzheimer’s disease (AD) is attracting attention. However, the mechanisms underlying the therapeutic efficacy of rTMS have not yet been elucidated, and few studies have systematically analyzed the stimulation parameters. In this study, we found that treatment with rTMS contributed to restoration of memory deficits by activating genes involved in synaptic plasticity and long-term memory. We evaluated changes in several intracellular signaling pathways in response to rTMS stimulation; rTMS treatment activated STAT, MAPK, Akt/p70S6K, and CREB signaling. We also systematically investigated the influence of rTMS parameters. We found an effective range of applications for rTMS and determined the optimal combination to achieve the highest efficiency. Moreover, application of rTMS inhibited the increase in cell death induced by hydrogen peroxide. These results suggest that rTMS treatment exerts a neuroprotective effect on cellular damage induced by oxidative stress, which plays an important role in the pathogenesis of neurological disorders. rTMS treatment attenuated streptozotocin (STZ)-mediated cell death and AD-like pathology in neuronal cells. In an animal model of sporadic AD caused by intracerebroventricular STZ injection, rTMS application improved cognitive decline and showed neuroprotective effects on hippocampal histology. Overall, this study will help in the design of stimulation protocols for rTMS application and presents a novel mechanism that may explain the therapeutic effects of rTMS in neurodegenerative diseases, including AD.

## Introduction

Transcranial magnetic stimulation (TMS) is a non-invasive brain stimulation technique that uses local magnetic field pulses to stimulate the cerebral cortex through an intact skull [1]. TMS creates an intense magnetic field by passing a strong current through an electromagnetic coil placed over the scalp [2]. The generated magnetic field passes through the skull unhindered, inducing microscopic currents in cortical tissue, which in turn stimulate brain cells [3, 4]. When a train of a single TMS pulse is applied continuously at a constant stimulus intensity, the procedure is referred to as repetitive TMS (rTMS). Repeated pulses may have a more lasting effect on improving brain function [5, 6]. Frequency refers to the stimulation rate of rTMS; stimulation below 1 Hz is classified as low frequency, and stimulation above 1 Hz is classified as high frequency [7]. In general, stimulation with high frequency rTMS increases cortical excitability, whereas stimulation with low frequency rTMS decreases excitability [8, 9]. Thus, stimulation frequency is an important parameter for cortical activity regulated by rTMS [10]. rTMS has been proven effective as a treatment for a variety of neurological and psychiatric disorders. Although treatment of depression was targeted first, research has expanded to include schizophrenia, post-traumatic stress disorder, insomnia, Alzheimer’s disease (AD), and Parkinson’s disease (PD) [11–17]. Although the therapeutic effects of rTMS on various brain diseases have been demonstrated, the molecular mechanisms by which rTMS restores brain function have not yet been elucidated.

AD is a neurodegenerative disorder characterized by decreased cognitive function, which includes behavioral disturbances and memory loss. The main neuropathological features of AD are amyloid plaques, entanglement of nerve fibers composed of hyperphosphorylated tau, and loss of neurons and synapses [18, 19]. In various neurodegenerative disorders, including AD, impaired long-term memory (LTM) is primarily caused by disruption of synaptic function and lack of LTMs, resulting in memory deficit and cognitive decline [20–22]. Transcriptional levels of genes associated with synaptic plasticity, learning, and memory processing, including immediate early genes such as c-Fos, JunB, activity-regulated cytoskeleton-associated protein (Arc), and early growth response gene-1 (Egr-1), are decreased in the hippocampus in AD patients and animal models [20, 23]. Targeting and manipulating the activity of genes involved in synaptic plasticity and LTM formation in AD patients could be a promising approach to slow or prevent disease progression.

Protein phosphorylation, a reversible post-translational modification, has been extensively studied and mainly regulates intercellular signaling pathways [24, 25]. Phosphorylation is an effective means of controlling cellular processes to regulate cellular functions, such as cell cycle progression, proliferation, differentiation, metabolism, cell survival, and apoptosis [26, 27]. The Janus kinase (JAK)/signal transducer and transcriptional activator (STAT), phosphatidylinositol 3-kinase (PI3K)/protein kinase B (Akt), and mitogen-activated protein kinase (MAPK) signaling pathways are controlled by protein phosphorylation. Activation of STAT3 and STAT5 results in upregulation of genes involved in cell survival, proliferation, and self-renewal, such as B-cell lymphoma 2 (Bcl-2), Bcl-xL, and c-Myc, and offers the advantage of inhibiting apoptosis [28, 29]. Akt is a potent apoptosis inhibitory protein. Phosphorylated Akt not only increases the expression of anti-apoptotic proteins but also inhibits activation of the apoptotic caspase pathway and poly(ADP-ribose) polymerase (PARP) cleavage, which is important for apoptosis [30, 31]. Activation of extracellular signal-regulated kinase 1/2 (ERK1/2) promotes cell survival using the same mechanism [32, 33]. The anti-apoptotic effect promoted by activation of these cellular signaling pathways extends to neuroprotection. cAMP-response element binding protein (CREB) activity is regulated by various protein kinases, and Akt and ERK1/2 pathways are upstream protein kinases involved in CREB activation [34]. In the brain, CREB is an important factor in neurogenesis, differentiation, proliferation, and cell survival [35]. CREB has also been proposed as an important regulator of learning and memory formation in the adult brain [36]. CREB contributes to the formation and consolidation of LTM by regulating the expression of target genes, such as c-Fos, c-Jun, brain-derived neurotrophic factor (BDNF), and Arc [37, 38]. For this reason, CREB activation is an attractive therapeutic target for neurodegenerative diseases aimed at improving memory [39].

Oxidative stress contributes to the pathogenesis of a variety of diseases including cancer, chronic inflammatory diseases, ischemic/reperfusion injury, cardiovascular disease, diabetes mellitus, and neurodegenerative diseases [40, 41]. The brain is highly susceptible to oxidative damage due to its abundance of polyunsaturated fatty acids and high oxygen metabolism. Therefore, oxidative damage has been studied as a central mechanism of neuropathology [42, 43]. Oxidative stress arises from excess formation of reactive oxygen species caused by disrupted redox homeostasis. Oxidative stress caused by uncontrolled or exogenous exposure damages DNA, proteins, lipids, and polysaccharides and, when cells exceed their ability to repair the damage, loss of function and cell death results [44]. Neuronal cell death is a pervasive feature of neurodegenerative disorders such as AD, PD, and Huntington’s disease [45]. Finding ways to prevent, inhibit, and ameliorate neuronal cell death is thus a promising treatment strategy. The non-radical form of hydrogen peroxide (H_2_O_2_) causes lipid peroxidation and damage to cellular components, leading to cell death [46]. H_2_O_2_ is often used to study cell death by mimicking oxidative stress in vitro.

Streptozotocin (STZ) is a methylated compound that, when metabolized, induces diabetes by targeted destruction of pancreatic beta cells [47]. Interestingly, intracerebroventricular (ICV) administration of STZ at low doses (< 1–3 mg/kg) that did not induce systemic diabetes resulted in cognitive impairment, oxidative stress, neurodegeneration, intracerebral glucose metabolism, cholinergic impairment, neuroinflammation, and abnormal phosphorylation of tau protein [48–50]. Therefore, STZ treatment is considered to induce neuropathological, biochemical, and behavioral features similar to AD. Administration of ICV-STZ in rodents is used as a non-transgenic model of AD. In particular, it is used to explore the etiology and molecular mechanisms of sporadic AD and to develop potential treatments [51, 52].

Expansion of the therapeutic application range of rTMS to various brain diseases as well as to depression is ongoing. However, the biological mechanisms underlying the therapeutic efficacy of rTMS have not been fully elucidated. The aim of this study was to investigate the effects of rTMS for application in the treatment of neurodegenerative diseases, particularly AD. We investigated whether rTMS treatment was effective in regulating gene expression related to synaptic plasticity and LTM. We also explored the cytoprotective effects of rTMS on inhibition of oxidative stress-induced neuronal cell death. Overall, we aimed to elucidate the effects of rTMS on the regulation of various signaling pathways involved in cell changes. In particular, for rTMS efficiency, various variables must be considered. Therefore, we focused on evaluating the effects of frequency, intensity, time, and duration in terms of application of rTMS, to determine optimal conditions. Finally, the therapeutic efficacy of rTMS for improvement of AD through cognitive enhancement and cytoprotective effects was verified using the STZ-induced sporadic AD model.

## Materials and Methods

### Chemicals and Reagents

H_2_O_2_ (30%) and crystal violet was purchased from Sigma (St. Louis, MO, USA). MTT reagent was purchased from Duchefa Biochemie (Haarlem, Netherlands). anti-p^Y701^-STAT1, anti-STAT1, anti-p^Y705^-STAT3, anti-STAT3, p^Y694^-STAT5, anti-STAT5, anti-p^T202/Y204^-p44/42 MAPK (Erk1/2), anti-p44/42 MAPK (Erk1/2), anti-p^T183/Y185^-SAPK/JNK and anti-SAPK/JNK, anti-p^T180/Y182^-p38, anti-p38, anti-p^S473^-Akt, anti-Akt, anti-P^T389^-p70S6 kinase, anti- p70S6 kinase, anti-p^S133^-CREB, anti-CREB were purchased from Cell Signaling Technology (Danvers, MA, USA). anti-Synaptophysin, anti-PSD95, anti-p^T205^-Tau, anti-p^S262^-Tau, and anti-Tau were purchased from ABclonal (Wuhan, China). anti-α-Tubulin was purchased from Abbkine (Wuhan, China). All other chemicals were obtained from Sigma-Aldrich (St. Louis, MO, USA).

### Cell Culture

Cells were maintained at 37 °C in a humidified atmosphere with 5% CO_2_. SH-SY5Y (human neuroblastoma cell line) and HT22 (mouse hippocampal neuronal cell line) were purchased from the Korean Cell Line Bank (Seoul, Korea) and American Type Culture Collection (Rockville, MD, USA), respectively. The cell lines were cultured in Dulbecco’s modified Eagle’s medium (DMEM, Hyclone, Logan, UT) supplemented with 10% heat-inactivated fetal bovine serum (FBS, Hyclone) and 1% penicillin/streptomycin solution (Capricorn Scientific GmbH).

### Application of rTMS

rTMS was performed using a Brain-Stim-A (REMED, Daejeon, Korea) with a figure-eight coil. The miniaturized rTMS device used was described previously [53]. The culture dish was placed 1 cm away from the center of the cross of a figure-eight–shaped magnetic coil. The high frequency stimulation protocol used in this study consisted of 10 Hz (inter-train interval of 26 s) for 4 s. The low frequency was applied as a continuous stimulation of 1 Hz.

To test the inhibitory effect of rTMS on neuronal cell death caused by exposure to oxidative stress, SH-SY5Y cells were treated with 10 Hz rTMS at 1.75 T three times a day for 30 min, for 2 days for a total of six times. Cells were subjected to oxidative stress by H_2_O_2_. After 48 h of initial stimulation with rTMS, cells were exposed to 400 µM H_2_O_2_ for an additional 12 or 24 h.

### RNA Extraction and Quantitative Real-Time PCR

Total RNA was extracted from SH-SY5Y cells using the RNAiso Plus reagent (Takara, Shiga, Japan) according to the manufacturer’s protocols. cDNA synthesis was performed with 1 µg of total RNA using a ReverTra Ace qPCR RT Master Mix (TOYOBO, Osaka, Japan). Quantitative real-time PCR was carried out using EvaGreen 2 × qPCR Mastermix (Applied Biological Materials, Richmond, BC Canada) on CFX Connect Real-Time PCR Detection System (Bio-Rad, Hercules, CA, USA). mRNA expression data were normalized using GAPDH expression as an internal standard. The sequences of the primers used for qPCR are presented in Table 1.

**Table 1:** Oligonucleotide sequences for the quantitative RT-PCR

Gene name		Sequence ( 5′ ≥ 3′)
*c-Fos (human)*	Forward	TGCAGCCAAATGCCGCAAC
	Reverse	TCGGTGAGCTGCCAGGATG
*c-Jun (human)*	Forward	GTCCTTCTTCTCTTGCGTGG
	Reverse	GGAGACAAGTGGCAGAGTCC
*JunB (human)*	Forward	GCACTAAAATGGAACAGCCCTT
	Reverse	GGCTCGGTTTCAGGAGTTTG
*Arc (human)*	Forward	ACAACAGGTCTCAAGGTTCCC
	Reverse	AGCCGACTCCTCTCTGTAGC
*Egr-1 (human)*	Forward	GGTCAGTGGCCTAGTGAGC
	Reverse	GTGCCGCTGAGTAAATGGGA
*NR4A1 (human)*	Forward	CCAAGTACATCTGCCTGGCTA
	Reverse	GACAACTTCCTTCACCATGCC
*NPAS4 (human)*	Forward	GCACTCGTGCAAGCACAC
	Reverse	AGAGACGCTACGTTCCTTTCC
*Homer1a (human)*	Forward	TTTGGTTGCTCGCTCCAC
	Reverse	TAAGGCTGCGGGTTCAAA
*GAPDH (human)*	Forward	CTGACTTCAACAGCGACACC
	Reverse	TAGCCAAATTCGTTGTCATACC

### Western Blotting

Cells were washed twice with ice-cold PBS, lysed with Triton lysis buffer containing phosphatase and protease inhibitors (1 mM PMSF, 1 mM EDTA, 0.2 mM Na_3_VO_4_, 0.5 mM NaF, and 1 µg/ml leupeptin) on ice for 30 min. Lysates were centrifuged at 13,000 rpm for 15 min at 4 °C, and supernatants were collected. Protein concentrations were measured with the Protein Assay Dye Reagent Concentrate (Bio-Rad, Hercules, CA, USA). Lysates were boiled with sample buffer containing β-mercaptoethanol for 5 min, and equal amounts of protein were separated on 6–13% SDS-PAGE followed by transferred to nitrocellulose blotting membranes (GE Healthcare Life Sciences, Chicago, IL, USA). Blots were blocked with 5% skim milk in TBS containing Tween 20 (TBS-T) for 1 h and incubated with primary antibodies overnight at 4 °C. The following day, blots were hybridized with horseradish peroxidase-conjugated secondary antibodies (Enzo Life Sciences, Farmingdale, NY, USA). Between each step, the blots were washed with TBS-T three times for 10 min each. The protein bands were detected by ECL chemiluminescence kit (Biomax, Seoul, Korea).

### Crystal Violet Staining

Cells were seeded into 60 mm plates at a concentration of 1.5 × 10^6^ cells/well and allowed to adhere for 24 h in 5% CO_2_ incubator at 37 °C. After TMS treatment, the plates were washed with PBS, and cells were stained with 0.5% crystal violet in H_2_O with 20% methanol and incubated for 20 min at room temperature with shaking.

### Cell Viability Analysis

Cell viability was assessed using the MTT assay. After treatment, the culture medium was removed and MTT solution (0.5 mg/ml) was added. Plates were incubated at 37 °C for 2 h. DMSO was used to dissolve the formazan crystals. Absorbance was measured with an ELISA reader (Tecan, Männedorf, Switzerland) at 570 nm. Data were expressed as percentage of control.

### IncuCyte Cytotox Green Assay

Cytotoxicity assays were performed using the IncuCyte Cytotox Green Dye (Essence BioScience, Ann Arbor, MI, USA) according to the manufacturer’s instructions. This cyanine nucleic acid dye stains dead cells. Normal cells are unaffected; damaged cells have increased cell permeability. When the reagent penetrates the nucleus and binds DNA, it emits fluorescence. After rTMS treatment, a medium containing IncuCyte Cytotox Green Dye adjusted to a final concentration of 250 nM was added to each well. Cells were treated with H_2_O_2_, imaged using the IncuCyte Live-Cell Imaging System (Sartorius, Göttingen Germany) and analyzed using IncuCyte 2021A software (Sartorius, Göttingen Germany). Green fluorescence was assessed 24 h after H_2_O_2_ treatment, and results were normalized to untreated controls.

### Apoptosis Analysis

Apoptosis assay was conducted using annexin V-FITC and Propidium Iodide (PI) detection kit (Becton Dickinson Bioscience, San Jose, CA) according to the manufacturer’s instructions. Briefly, after the required treatment, SH-SY5Y cells were washed twice with ice-cold PBS, collected and subsequently resuspended in 100 µl binding buffer. Cells were stained with 5 µl of annexin V-FITC and 5 µl of PI in the dark at room temperature for 15 min. A total of 400 µl binding buffer was added, and the cells were analyzed by flow cytometry (BD LSRFortessa). annexin V-positive cells were considered to be apoptotic cells.

### Animal Experiments

Experiment protocols were reviewed and approved by the Animal Care Committee of the Catholic Kwandong University (No. 2019–005). A total of 15 male Sprague–Dawley rats (weighing 250 ± 30 g, 7-week-old) were purchased from Samtaco (Samtaco Bio Korea, Korea). The animals were placed in a quiet room at an ambient room temperature of 22 ± 3 °C, relative humidity of 55 ± 10%, and a 12 h dark/light cycle with food and water ad libitum. All animals were acclimatized for 7 days prior to the start of experiment.

Rats were randomly divided into three groups: sham-operated, STZ, and STZ + rTMS. The animals were anesthetized with intraperitoneal (i.p.) injections of sodium pentobarbital (40 mg/kg; Sigma Chemical Co., St. Louis, MO, USA), and the head was placed in a stereotaxic frame. To target the hippocampal region, burr holes were drilled in both lateral ventricles of the skull with the following coordinates: 0.8 mm posterior to the bregma, 1.5 mm lateral to the sagittal suture, and 3.6 mm beneath the surface of the brain. Rats were injected bilaterally with ICV-STZ (total volume of 4 µl at a rate of 1 µl/min) through a hole in the skull using a 26 gauge microneedle. STZ was freshly dissolved in saline immediately prior to application. For rats in the sham surgery group, the same operation was performed, but saline was injected.

To evaluate the effects of rTMS in the STZ-induced AD model, rats were acclimated and stabilized for 1 week after STZ injection, and then stimulated with rTMS at 10 Hz for 20 min. To prevent the animal from moving during the experiment, it was fixed using a holder, and stimulation was performed in the awake state without anesthesia. rTMS was performed by floating 2 cm away from the cerebral cortex of the rat. Stimulation was applied at the same time for 20 min, once a day, five times a week for 4 weeks.

### Y-Maze Spontaneous Alternation

The Y-maze test was performed the day after the experiment was completed. Rats were tested for spontaneous alternation in a Y-maze with three arms made of black polyvinyl plastic. The instrument consists of three branches and an equilateral triangular central area. Each arm is 42 cm long, 8 cm wide, and 21 cm high, and the folding angle between the three arms is 120°. After setting each branch to A, B, and C, the test rat was carefully placed on one arm and allowed to explore freely. The movement path of the arm into which the test rat entered was recorded for 8 min, and the number of times the tail of the rat entered completely was counted for each arm. The total number and order of arms entered by the rat was recorded and assigned a score of one when it entered three different arms without overlapping (actual alternation). Alternating behavior was defined as successive entry into three different arms and was calculated with the following equation: spontaneous alternations (%) = number of alternations/maximum possible alternations (total number of arms entered − 2) × 100.

### Tissue Sample Preparation

Rats were sacrificed immediately after the final experiment, and brain tissue samples were prepared. Following anesthesia with Zoletil 50, rats were transcardially perfused with 50 mM phosphate-buffered saline, and brains were fixed with a freshly prepared solution of 4% paraformaldehyde in 100 mM phosphate buffer. After brain harvest, the perfused samples were post-fixed overnight at 4 °C using the same fixative and transferred to a 30% sucrose solution to prevent freezing. Coronal Sections (40 μm thick) were generated using a freezing microtome.

### Nissl Staining

To observe the brain hippocampus, nissl staining was performed on the brain sections mounted on the slide using cresyl violet. Brain sections were immersed in xylene twice for 3 min each, and then hydrated using an alcohol concentration gradient (80%, 90%, 100%, 3 min each). After dehydration using an alcohol gradient (70%, 90%, 100%, 1 min each), the sections were cleaned with xylene, mounted with Permount (Fisher Scientific, USA) and covered with a cover glass.

### Statistical Analysis

The data were analyzed using Microsoft Excel 2016 software and GraphPad Prism 5 (GraphPad Software, Inc, La Jolla, CA, USA). The results were expressed as the mean ± SD from least three independent experiments. Statistical significance was calculated using an un-paired Student’s *t*-test or one-way ANOVA followed by Tukey’s multiple comparisons test. A *P* value of < 0.05 was considered significant for all statistical analyses.

## Results

### rTMS Elevated Expression of Genes Related to Synaptic Plasticity and Long-Term Memory

**Fig. 1:**
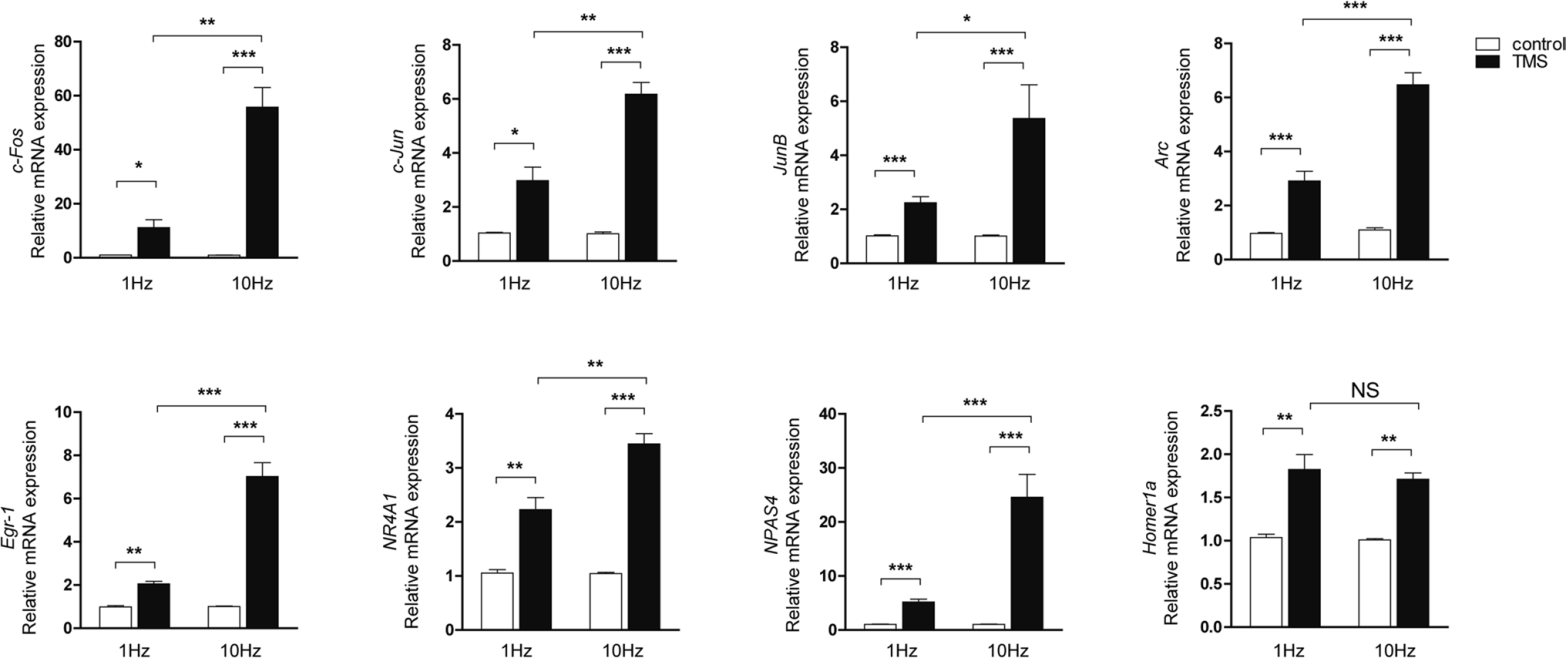
rTMS treatment increases the expression of genes related to synaptic plasticity and long-term memory. SH-SY5Y cells were treated with low frequency (1 Hz) or high frequency (10 Hz) rTMS at 1.75 T for 30 min. mRNA levels of *c-Fos*, *c-Jun*, *JunB*, *Arc*, *Egr-1*, *NR4A1*, *NPAS4*, and *Homer1a* were analyzed by quantitative real-time PCR. GAPDH was used as a standard. Data are shown as mean ± SD (*n* = 3). NS, not significant. *P < 0.05, **P < 0.01, ***P < 0.005, determined by Student’s *t*-test

rTMS, a non-invasive brain stimulation technique, has been used to treat various neurological diseases. Although rTMS has recently been proposed as a promising treatment to improve memory and learning ability, more research is needed to understand the role of rTMS in ameliorating AD. Disruption of synaptic function and deficits in LTM are major causes of several neurodegenerative disorders, including AD, which is characterized by memory deficits and cognitive decline [20–22]. It has been suggested that upregulation of gene expression related to synaptic plasticity, learning, and memory may improve LTM and protect against the negative cognitive consequences of neurodegeneration [23]. Specifically, CREB positively regulates neuroplasticity, memory formation and enhancement, and learning in the adult brain [34]. Previous studies have reported that rTMS treatment has a positive effect on CREB activation [54]. Upregulated expression of target genes such as *c-Fos, JunB, Arc, Egr-1*, and *NR4A1* by activation of CREB improves synaptic plasticity and LTM, contributing to memory consolidation, and enhancement [55, 56]. We also selected and further investigated other genes involved in improving memory and learning [57, 58]. In this study, we tested the hypothesis that regulation of genes related to synaptic plasticity and LTM could improve memory and learning ability in rTMS treatment. To investigate the effect of rTMS on LTM-related gene expression, SH-SY5Y cells were treated with low or high frequency rTMS for 30 min at 1.75 T intensity. Treatment with rTMS significantly increased synaptic plasticity and LTM-associated gene mRNA levels, compared to untreated controls. In addition, mRNA expression analysis further showed higher upregulation by high frequency rTMS treatment when low and high frequency rTMS treatment groups were compared (Fig. 1). These data suggest that increased synaptic plasticity and expression of LTM-related genes is one mechanism explaining the effects of rTMS stimulation in the treatment of diseases associated with decreased memory and learning.

### Stimulation of rTMS Activates STAT, MAPK, Akt/p70S6K, and CREB Signaling Pathways

**Fig. 2:**
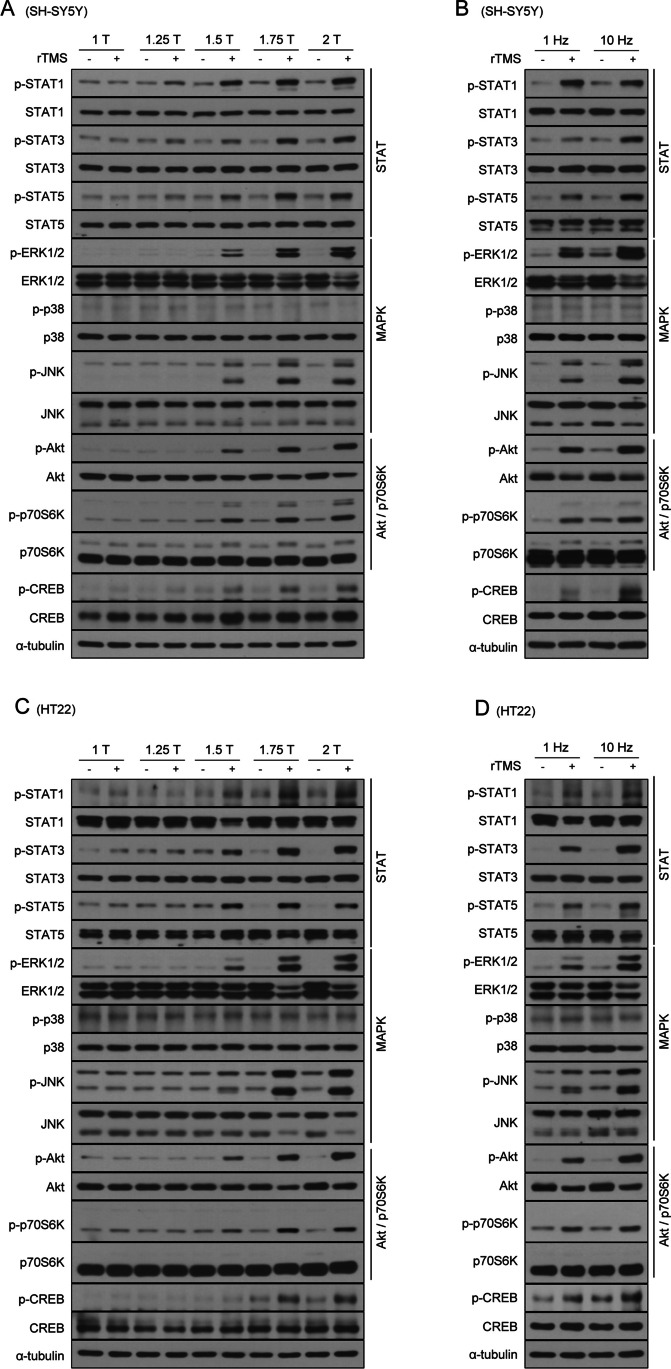
Treatment with rTMS induces activation of STAT, MAPK, Akt/p70S6K, and CREB signaling pathways. **A** SH-SY5Y cells were treated with 10 Hz rTMS for 30 min at the indicated intensity. **B** SH-SY5Y cells were treated with low frequency (1 Hz) or high frequency (10 Hz) rTMS for 30 min at 1.75 T. **C** HT22 cells were treated with 10 Hz rTMS for 30 min at the indicated intensity. **D** HT22 cells were treated with low frequency (1 Hz) or high frequency (10 Hz) rTMS for 30 min at 1.75 T. Cell lysates were subjected to immunoblotting with the indicated antibodies. α-tubulin was used as a standard

The major upstream regulator of LTM-associated genes identified in Fig. 1 is CREB [55, 56]. The transcriptional activity of CREB is induced by phosphorylation and plays an important role in the regulation of various cellular functions, including growth and proliferation [34]. Protein phosphorylation is an important mechanism of action in which extracellular and intracellular signals affect gene expression in the nucleus. Phosphorylation plays an important role in the regulation of many cellular functions, including the cell cycle, proliferation, growth, survival, and apoptosis. It also regulates signal transduction, transcription, and protein function [59]. CREB is phosphorylated by upstream regulators in ERK and Akt signaling pathways [34]. The neurobiological mechanisms underlying the effects of rTMS remain unclear. Therefore, we investigated whether rTMS stimulation could affect the phosphorylation of key proteins not only in CREB signaling but also in other signaling pathways such as STAT (STAT1, STAT3, STAT5), MAPK (ERK, JNK, p38), and Akt/p70S6K. We also set out to determine the optimal parameters. To investigate the phosphorylation of intracellular signaling pathways by the intensity of rTMS treatment, SH-SY5Y cells were treated with rTMS in an intensity-dependent manner. The time and frequency were kept at 30 min and 10 Hz, respectively, which did not induce cell damage. rTMS treatment significantly upregulated the phosphorylation of STAT1, STAT3, STAT5, ERK, JNK, Akt, p70S6K, and CREB. Increased phosphorylation was observed in cells treated with rTMS at an intensity of 1.5 T for 30 min, with an apparent maximum at 1.75 T (Fig. 2A). Since one of the important parameters for optimal rTMS treatment is frequency, we examined the effects of low (1 Hz) and high (10 Hz) frequency on activation of signaling pathways. Both low and high frequency significantly increased phosphorylation levels in SH-SY5Y cells. In addition, high frequency rTMS induced markedly higher levels of protein phosphorylation, compared with low frequency rTMS (Fig. 2B). To confirm the effects of rTMS on protein phosphorylation in other neuronal cells, HT22 cells, a mouse hippocampal neuronal cell line, were treated with rTMS. Similar results were obtained to those of SH-SY5Y cells (Fig. 2C, D). Taken together, these data suggest that rTMS activates various signaling pathways through induction of protein phosphorylation in neurons. In addition, regulation of the signaling pathway by rTMS showed a clear effect at the 1.75 T intensity, and high frequency rTMS was more effective than low frequency rTMS under the same time and intensity conditions.

### The Increase in Protein Phosphorylation Occurs Faster and Lasts Longer with High Frequency rTMS

**Fig. 3:**
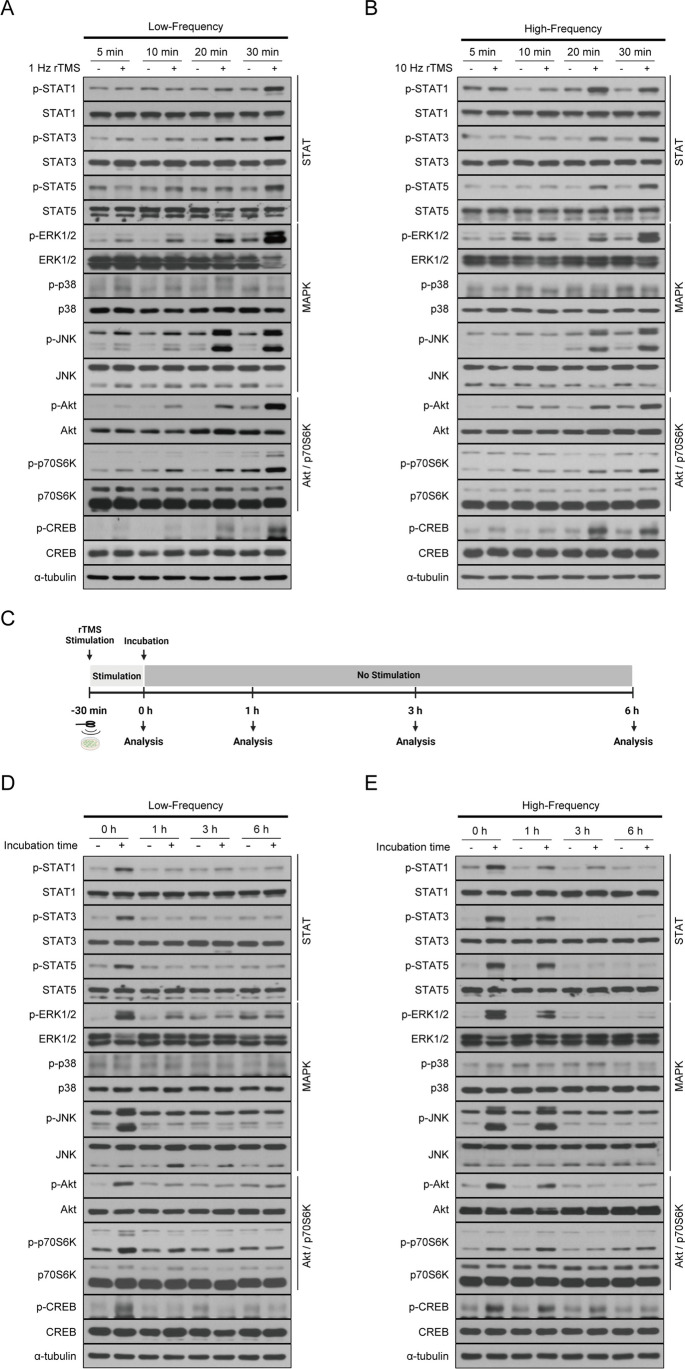
Induction of protein phosphorylation by rTMS treatment is faster and longer lasting at high frequencies. **A** SH-SY5Y cells were treated with low frequency (1 Hz) rTMS at 1.75 T for the indicated times. **B** SH-SY5Y cells were treated with high frequency (10 Hz) rTMS at 1.75 T for the indicated times. **C** Schematic overview of experimental design. Image created with BioRender.com. **D** SH-SY5Y cells were treated with low frequency (1 Hz) rTMS at 1.75 T for 30 min and then incubated for the indicated time period without stimulation. **E** SH-SY5Y cells were treated with high frequency (10 Hz) rTMS at 1.75 T for 30 min and then incubated for the indicated time period without stimulation. **A**–**E** Cell lysates were subjected to immunoblotting with the indicated antibodies. α-tubulin was used as a standard

We next investigated the effect of intracellular signaling pathway activation on the stimulation time of rTMS. To compare further the effects of frequency of rTMS on the induction of protein phosphorylation, SH-SY5Y cells were treated with low (1 Hz) or high (10 Hz) frequency rTMS in a time-dependent manner. Upregulation of p-ERK, p-JNK, p-Akt, and p-p70S6K was observed at both low and high frequency after 20 min in rTMS-exposed cells. Phosphorylation of STAT was increased at 20 min with high frequency while this was delayed somewhat at low frequency (Fig. 3A, B). Notably, the level of p-CREB increased at 20 min in high frequency rTMS-treated cells but not until 30 min in low frequency rTMS-treated cells (Fig. 3A, B). Next, we investigated how long the increase in protein phosphorylation induced by rTMS stimulation persisted. Cells were treated with low or high frequency rTMS at an intensity of 1.75 T for 30 min, then further incubated without stimulation at 37 °C (Fig. 3C). The level of phosphorylation increased by low frequency rTMS treatment returned to control levels within 1 h (Fig. 3D). On the other hand, in the high frequency rTMS group, phosphorylation levels gradually decreased for 3 h after stimulation (Fig. 3E). These findings suggest that phosphorylation induced by high frequency rTMS treatment lasts longer than that of low frequency rTMS. Moreover, the increased phosphorylation due to rTMS stimulation returns to a steady state over time, suggesting that rTMS stimulation does not disrupt the regulatory function of the cellular signaling pathway network. These results collectively suggest that both low and high frequency rTMS activates signaling pathways in neurons, but high frequency rTMS induced a stronger and longer lasting effect, resulting in higher therapeutic efficacy.

### Finding Appropriate Stimulation Conditions Will Be Essential to Prevent Cell Death Due to Excessive rTMS Treatment

**Fig. 4:**
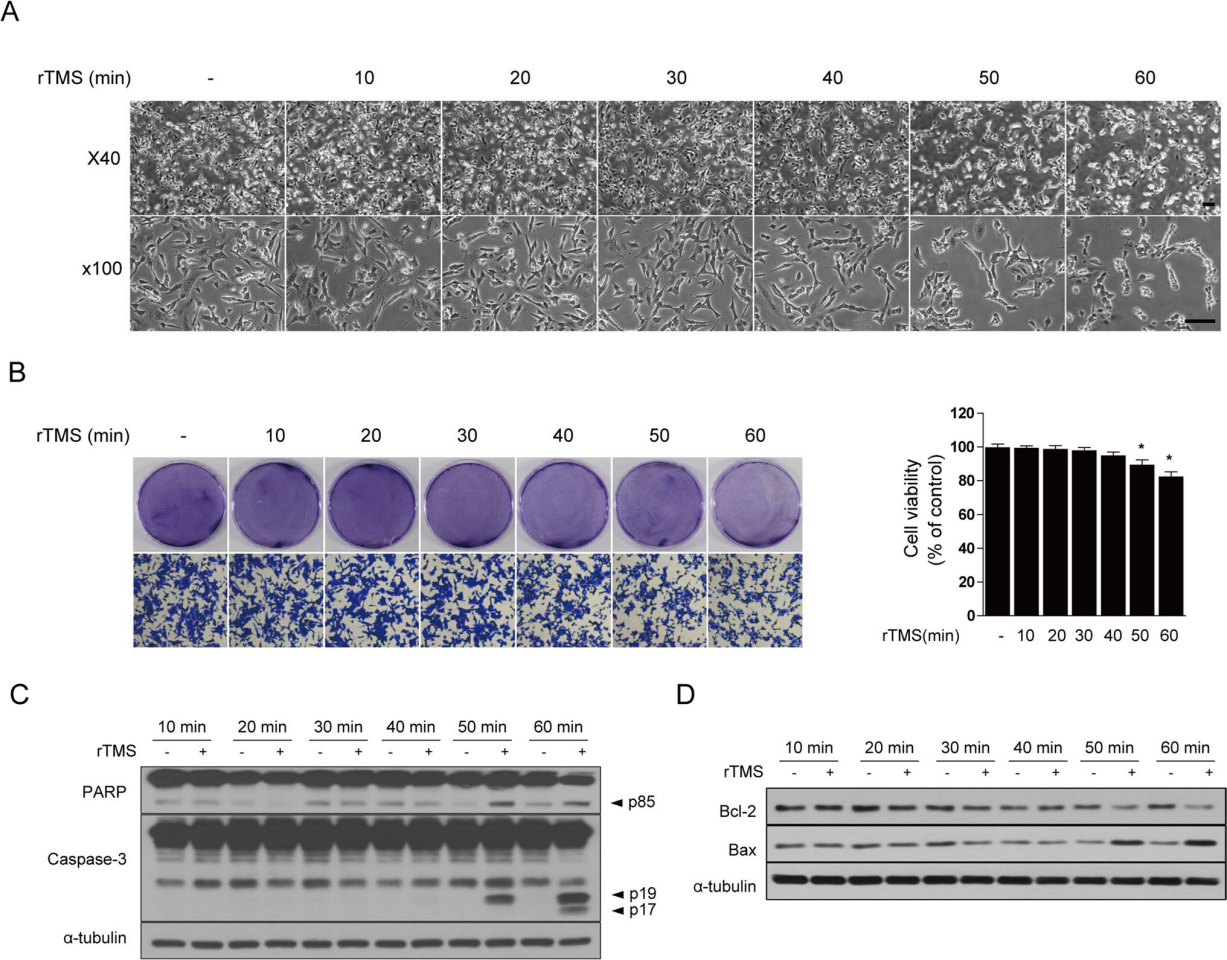
Excessive rTMS treatment causes neuronal cell damage and death. SH-SY5Y cells were treated with 10 Hz rTMS at 2 T for the indicated times. **A** Morphology was observed using a phase contrast inverted microscope. Scale bar, 100 µM. **B** Cell viability was determined by crystal violet staining. Representative images are shown on the left, and quantification is shown on the right. **C**, **D** Cell lysates were subjected to immunoblotting with the indicated antibodies. α-tubulin was used as a standard. Values are shown as mean ± SD of three replicates. *P < 0.05 vs. untreated control by Student’s *t*-test

rTMS not only promotes cell proliferation and survival but also inhibits apoptosis and loss in damaged neuronal cells [54, 60]. Imbalanced or excessive stimulation of rTMS can lead to cell damage and death, resulting in side effects and pain. Therefore, we conducted an in vitro study to evaluate the appropriate time to apply rTMS with a focus on efficacy and safety. SH-SY5Y cells were treated with rTMS at various time points with an intensity and frequency of 2 T and 10 Hz, respectively. The intensity and frequency values were set as the maximum values used in this study. rTMS-induced apoptotic cell death was detected by morphological changes, observed by inverted phase contrast microscopy. Application of rTMS induced cell loss after 50 min of treatment. In addition, the cells appeared rounded and detached, and degenerated cells were observed (Fig. 4A). Next, we used crystal violet to measure cytotoxic effects. After rTMS treatment, cell viability started to decrease significantly from 50 min (Fig. 4B). To confirm further the apoptotic effects of rTMS, expression levels of apoptosis-associated proteins were examined by western blotting. At 50 min, expression of pro-apoptotic proteins such as cleaved caspase-3, cleaved PARP, and Bax was increased, whereas expression of anti-apoptotic proteins such as Bcl-2 was decreased (Fig. 4C, D). Thus, our results showed that treatment with 10 Hz rTMS at 2 T can be applied in vitro for up to 40 min without cell damage and that excessive rTMS stimulation can induce apoptosis. In the future, it will be important to determine stimulation conditions suitable for rTMS treatment.

### rTMS Treatment Reduces Oxidative Stress-Induced Neuronal Cell Death and Exerts a Cytoprotective Effect

**Fig. 5:**
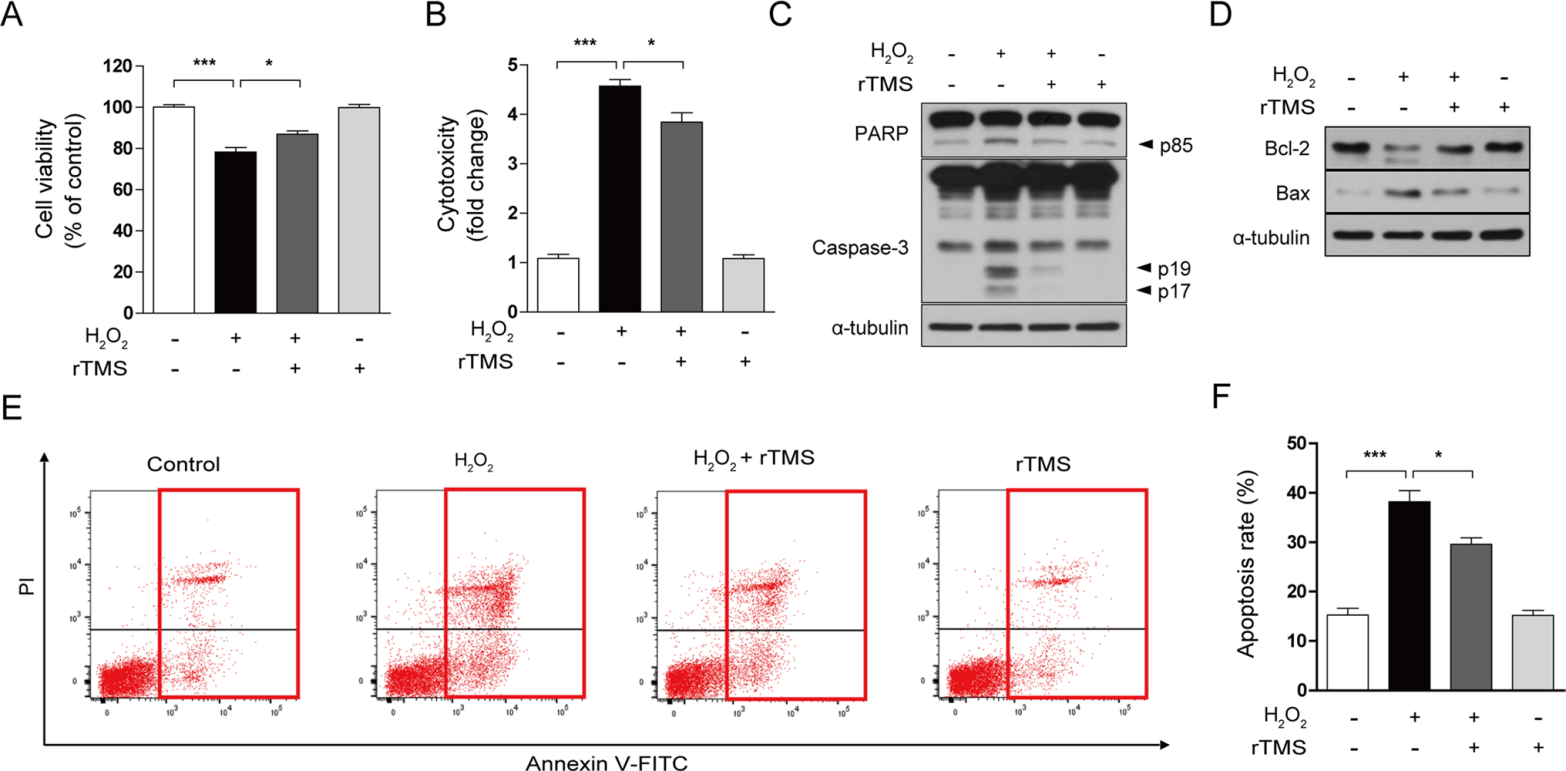
rTMS treatment reduces H_2_O_2_-induced neuronal cell death. SH-SY5Y cells were treated with 10 Hz rTMS at 1.75 T for 30 min, three times a day for 2 days for a total of six times. Then, 48 h after the initial rTMS stimulation, cells were treated with 400 µM H_2_O_2_ for an additional 12 or 24 h. **A** Cell viability was determined by MTT assay. **B** Dead cells were measured by staining with IncuCyte CytoTox Green Reagent. **C**, **D** Cell lysates were subjected to immunoblotting with the indicated antibodies. α-tubulin was used as a standard. **E**, **F** Apoptotic cells were detected using annexin V-FITC/PI staining and analyzed by flow cytometry. Annexin V-positive cells were considered apoptotic. All data are expressed as mean ± standard deviation of three replicates, and representative data are shown. *P < 0.05, **P < 0.01, ***P < 0.005, determined by Student’s *t*-test

Phosphorylation plays an important role in cell growth, survival, and maintenance, and contributes to neuroprotection and restoration of function by activating signals that prevent cell death and defend against damage [35, 61, 62]. Oxidative stress, one of the primary causes of degenerative brain disease, is known to damage nerve cells and induces cell death. In this study, we investigated whether rTMS exerts cytoprotective effects against cell damage and death induced by H_2_O_2_ exposure. SH-SY5Y cells were treated with 10 Hz rTMS at 1.75 T for 30 min, three times a day for 2 days for a total of six times. At 48 h after the first stimulation, cells were exposed to 400 µM H_2_O_2_ for another 12 h or 24 h. Analysis of cell viability with the MTT assay showed a decrease in the group exposed to H_2_O_2_, indicating cytotoxicity was induced by oxidative damage (Fig. 5A). On the other hand, rTMS treatment significantly increased cell viability, compared to the H_2_O_2_ alone group (Fig. 5A). To evaluate further the protective effect of rTMS, cytotoxicity was measured using IncuCyte CytoTox Green reagent, which stains dead cells. Treatment with rTMS significantly reduced the increase in cytotoxicity caused by H_2_O_2_ exposure (Fig. 5B). To evaluate the inhibition of apoptosis observed following rTMS treatment, we analyzed the expression of apoptosis-related proteins and performed annexin V/PI staining. In response to H_2_O_2_, protein expression of cleaved PARP, cleaved caspase-3, and Bax increased, while expression of Bcl-2 decreased; these effects were inhibited by rTMS stimulation (Fig. 5C, D). rTMS treatment also reduced the percentage of annexin V-positive cells (Fig. 5E, F). Thus, rTMS treatment reduces oxidative stress-induced cell death. This neuroprotective effect of rTMS highlights its potential for treatment of neurodegenerative diseases.

### rTMS Attenuates STZ-Induced Neuronal Cell Death and AD-like Pathology

**Fig. 6:**
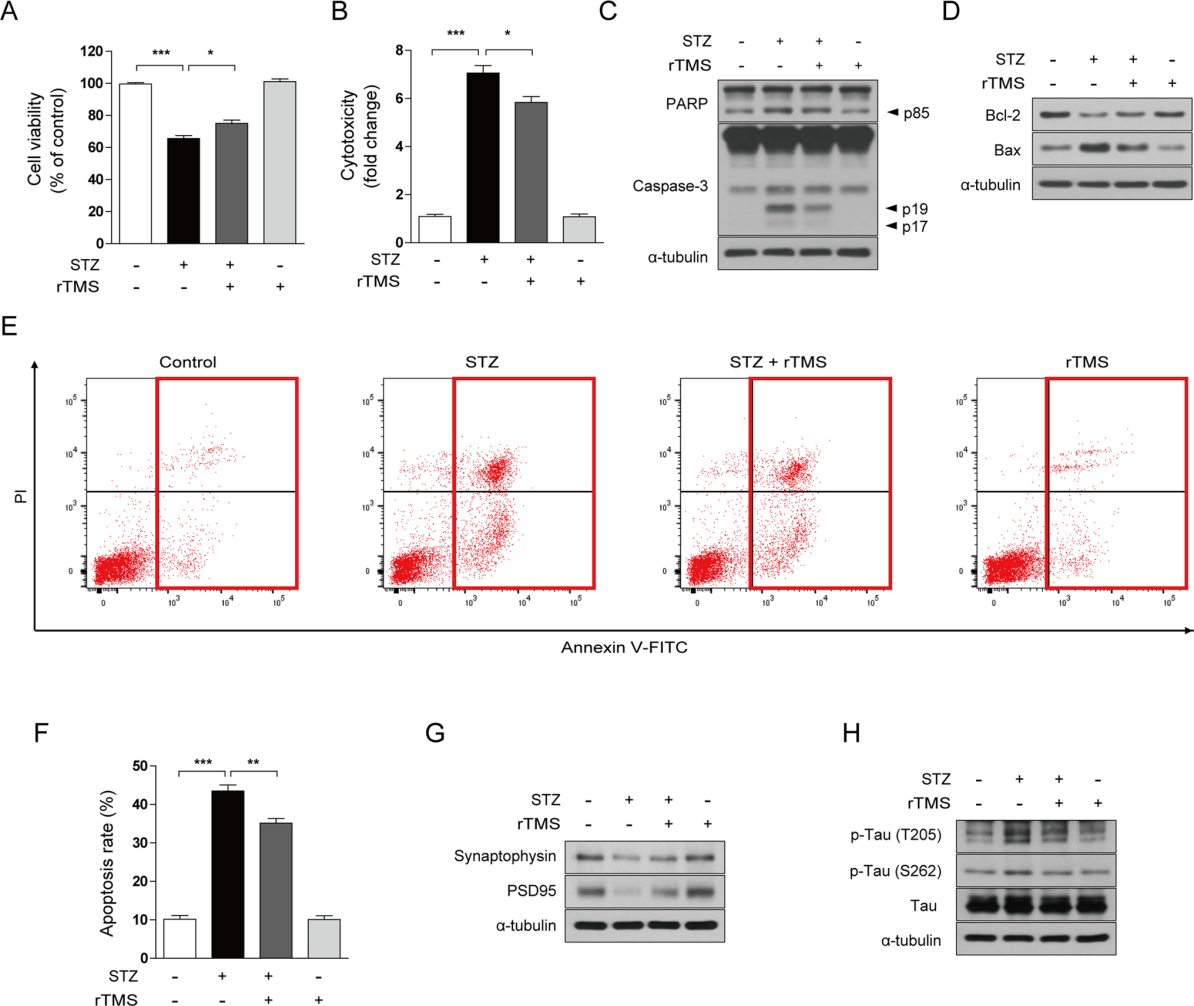
rTMS treatment alleviates STZ-induced neuronal cell death and AD-like pathology. SH-SY5Y cells were treated with 10 Hz rTMS at 1.75 T for 30 min, three times a day for 2 days for a total of six times. Then, 48 h after the initial rTMS stimulation, cells were treated with 5 mM STZ for an additional 12 or 24 h. **A** Cell viability was determined by MTT assay. **B** Dead cells were measured by staining with IncuCyte CytoTox Green Reagent. **C**, **D**, **G**, **H** Cell lysates were subjected to immunoblotting with the indicated antibodies. α-tubulin was used as a standard. **E**, **F** Apoptotic cells were detected using annexin V-FITC/PI staining and analyzed by flow cytometry. Annexin V-positive cells were considered apoptotic. All data are expressed as mean ± standard deviation of three replicates, and representative data are shown. *P < 0.05, **P < 0.01, ***P < 0.005, determined by Student’s *t*-test

Next, we investigated the effects of rTMS in the STZ-induced model of sporadic AD. STZ not only induces cell death in a cellular model but also promotes synaptic loss and tau hyperphosphorylation [63, 64]. To investigate the effects of rTMS on protection of STZ-induced cell death, SH-SY5Y cells were pretreated with rTMS and exposed to STZ. STZ treatment in SH-SY5Y cells decreased viability as measured by MTT assay and increased cell death (Fig. 6A, B). These results demonstrate STZ induces cytotoxicity and cell death. On the other hand, the effects of STZ were significantly antagonized by rTMS pretreatment (Fig. 6A, B). We also evaluated changes in the expression of apoptosis-related proteins and annexin V/PI staining, to determine the protective effects of rTMS against STZ-induced apoptosis. Expression of cleaved caspase-3, cleaved PARP, and Bax was increased by STZ treatment, whereas Bcl-2 expression was decreased (Fig. 6C, D). In addition, STZ treatment increased the percentage of annexin V-positive cells (Fig. 6E, F); these effects were mitigated by rTMS treatment (Fig. 6C–F). Next, we investigated whether rTMS treatment could inhibit AD pathologies such as STZ-promoted synaptic loss and aberrant phosphorylation of tau protein. STZ decreased expression of the synaptic markers synaptophysin and postsynaptic density protein 95 (PSD95) and upregulated phosphorylation of tau. However, rTMS treatment significantly ameliorated the STZ-induced effects (Fig. 6G, H). Thus, rTMS treatment alleviates STZ-induced apoptosis and AD-like pathology in neuronal cells.

### rTMS Application Ameliorates Cognitive Impairment and Exhibits Cytoprotective Effects in ICV-STZ Rats

**Fig. 7:**
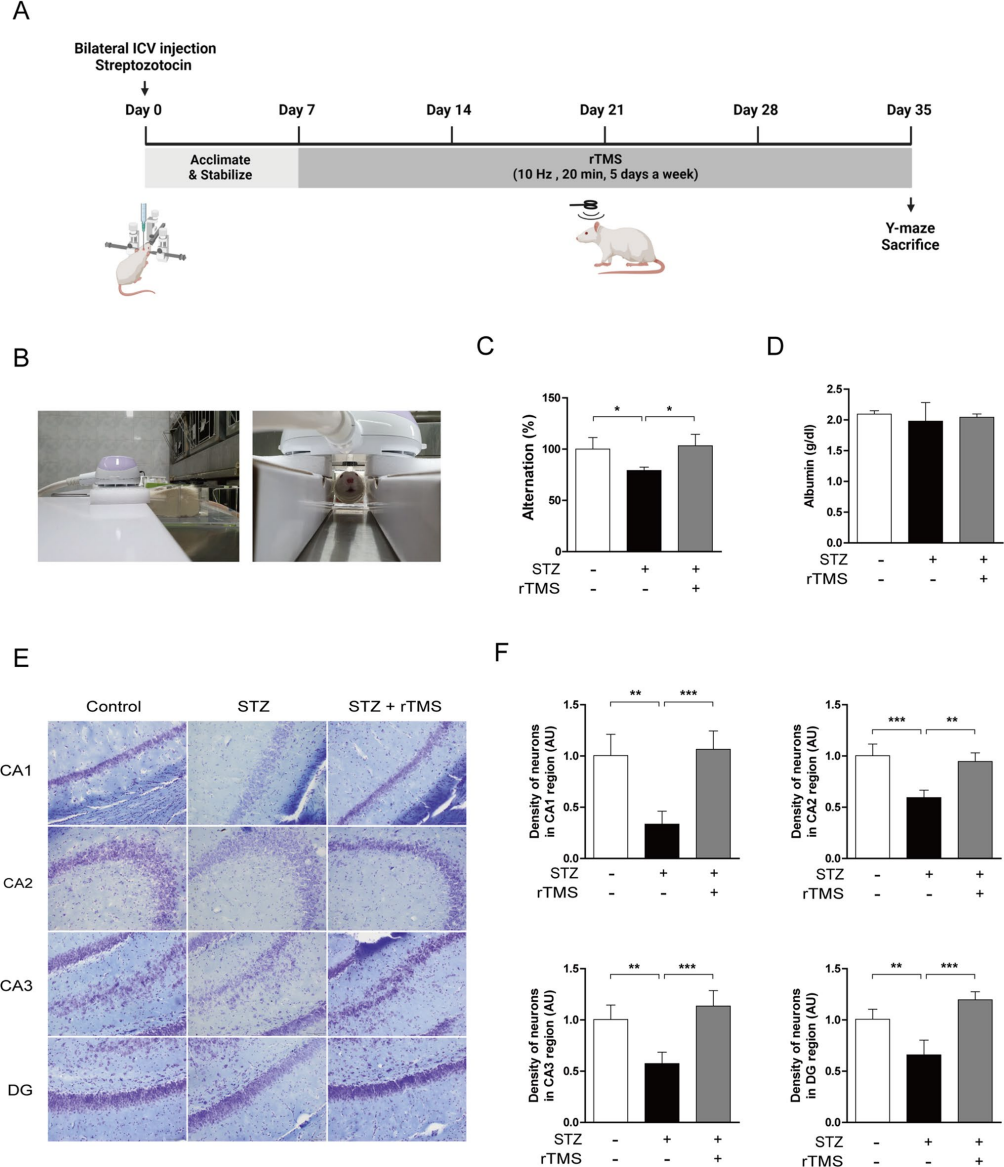
rTMS ameliorates memory deficit behavioral disorders and neuronal cell death in ICV-STZ rats. Rats were injected ICV with STZ (1.5 mg/kg, saline 5 µl). After 7 days of STZ administration, the rTMS application group was treated with high frequency (10 Hz) rTMS for 20 min a day, 5 days a week, for a total of 4 weeks. **A** Experimental design for animal experiments. Image created with BioRender.com. **B** Photograph of immobilized rats for rTMS application in the awake state. **C** Behavioral experiment on spontaneous alternation using the Y-maze. **D** Hepatic injury was assessed by measuring serum levels of albumin. **E** Representative images of Nissl staining in the CA1, CA2, CA3, and DG regions of the hippocampus. **F** Quantification of Nissl stain mean intensity in the CA1, CA2, CA3, and DG regions of the hippocampus. All data are presented as mean ± standard deviation (*n* = 4). *P < 0.05, analyzed by one-way ANOVA followed by Tukey’s multiple comparisons test

We further confirmed in animal models that rTMS has a cognitive enhancing effect and can be used as a therapeutic agent for AD. ICV injection of STZ is a commonly used research model that mimics the pathology of sporadic AD in humans. Low-dose ICV injections of STZ, which do not induce diabetes, have been shown to induce AD-like pathological and behavioral features, such as learning and memory impairments, oxidative stress, neurodegeneration, brain insulin signaling abnormalities, cholinergic disorders, and tau hyperphosphorylation [49, 50]. To evaluate whether rTMS treatment in ICV-STZ rats improved cognitive impairment, high frequency (10 Hz) rTMS was applied 20 min a day, 5 days a week for a total of 4 weeks (Fig. 7A). Rats were immobilized in the awake state without anesthesia, and only the skull was stimulated with rTMS (Fig. 7B). We investigated the effects of rTMS on the improvement of memory impairment in ICV-STZ rats using the Y-maze test. Memory impairment in ICV-STZ rats was improved by rTMS treatment (Fig. 7C). Hepatotoxicity was evaluated by measuring serum albumin; there were no significant changes with STZ and rTMS (Fig. 7D). We performed Nissl staining to evaluate histopathological differences in the rat hippocampus. Changes in hippocampal cornu ammonis (CA) 1, CA2, CA3, and dentate gyrus (DG) regions responsible for cognitive functions of the hippocampus were observed. Neurons in the hippocampus of the ICV-STZ group were misaligned, intercellular spaces were enlarged, cells were lost, and cell density was reduced (Fig. 7E, F). In the rTMS-treated group, similar to the control group, neurons were clearly arranged in a regular structure, and cell loss was not observed (Fig. 7E, F). These results suggest that application of rTMS in an ICV-STZ-induced sporadic AD induction model improves cognitive function and has neuroprotective effects on hippocampal histology. Therefore, we propose that rTMS could be applied as a strategy for treatment of AD.

## Discussion

rTMS, a non-invasive therapeutic approach utilizing electrical stimulation, is gaining attention as a treatment modality for brain disorders due to its painless nature and minimal side effects. In vitro experiments have been attempted to elucidate the molecular mechanisms underlying the effects of rTMS. Previous in vitro studies using standard rTMS devices were performed outside the incubator; these experiments were limited as they did not have ideal conditions for cell culture. In this study, a miniaturized rTMS device was applied, allowing for rTMS treatment while culturing inside the incubator. Previous clinical trials for depression have reported that the therapeutic effects of miniaturized rTMS did not differ from those of standard rTMS [53]. This study is the first report of the action of rTMS in an in vitro experiment with established stable culture conditions.

Recent research has extended the application of rTMS for treatment of a wide range of neurological diseases, among which the therapeutic application of rTMS is of particular interest in neurodegenerative diseases such as AD. rTMS treatment has been shown to induce significant improvement in AD patients with cognitive decline [65]. Furthermore, application of rTMS in AD animal models resulted in increased BDNF expression levels, decreased BACE1 expression levels, and decreased amyloid-β accumulation [66, 67]. However, studies on the specific mechanism of the therapeutic effects of rTMS in the treatment of AD are lacking. Synaptic dysfunction and LTM impairment are highly correlated with cognitive decline in AD patients [20–22]. This suggests that targeting synaptic plasticity and LTM formation in the hippocampus could be a promising approach for the treatment of AD. Therefore, we investigated whether rTMS treatment could modulate the expression of genes involved in synaptic plasticity and LTM formation in neurons. Our results suggest that rTMS increases the transcription of genes involved in synaptic plasticity, learning, and memory processing in neurons, contributing to memory enhancement and learning ability. High frequency rTMS has been reported to have a higher therapeutic effect and cognitive improvement, compared to low frequency rTMS in experimental models of cognitive impairment such as AD, consistent with our results [66].

The transcription of genes involved in synaptic plasticity and LTM is regulated by CREB, which is activated primarily by phosphorylation. In the present study, we screened several signaling pathways to elucidate the mechanisms regulated by rTMS and found that rTMS treatment increased phosphorylation of components of the STAT, MAPK, and Akt/p70S6K signaling pathways. The majority of rTMS research has focused on elucidating its efficacy and therapeutic action as an antidepressant, and BDNF is known to be involved. Previous studies have shown that rTMS treatment increases the expression of BDNF and that decreased BDNF levels in depression are normalized after rTMS treatment. These results were verified by analyzing patient serum levels and the brain tissue of experimental animals [68, 69]. CREB, activated by its upstream regulator ERK1/2 or Akt, acts as a transcription factor, increasing expression of its downstream target BDNF. Therefore, ERK/CREB/BDNF and Akt/CREB/BDNF signaling have also attracted attention in rTMS studies. We showed that treatment with rTMS upregulates phosphorylation of ERK, Akt, and CREB. Results consistent with our research have been previously reported [70]. Notably, as a result of screening the effects of rTMS on various signaling pathways, we found for the first time that rTMS treatment activates STAT signaling (STAT1, STAT3, STAT5) in neurons. Activation of Akt/p70S6K signaling was also observed. The importance of systematic investigation of the influence of various stimulation parameters to characterize the neurobiological effects of rTMS has been emphasized. Based on previous studies related to rTMS, we revealed intensity, frequency, time, and duration as important parameters of rTMS. Phosphorylation levels were analyzed to evaluate the effect of each parameter and to determine the optimal combination of parameters. We showed that 1.5–2 T intensity and 20–30 min were within the effective range of rTMS and found that high frequency rTMS exerted a greater influence than low frequency rTMS. Importantly, our results suggest that treatment with 10 Hz rTMS for 30 min at 1.75 T is optimal. Phosphorylation by rTMS was sufficiently induced by stimulation for 30 min, and then returned to the basal state when the stimulation was removed. Thus, the phosphorylated proteins regulate expression of target genes and then return to normal levels, suggesting rTMS does not impair cellular function. This study has some limitations as it was performed in an in vitro system. It is difficult to design an experiment by changing various variables in an animal model or clinical trial and analyze it systematically. Therefore, our results may provide basic guidelines when designing an optimal protocol for the therapeutic use of rTMS.

One of the most important aspects for therapeutic application of rTMS is determining control conditions to ensure stability. rTMS treatment at 2 T and 10 Hz resulted in cell loss and death at 50 min. These results suggest that rTMS overstimulation may pose a risk. Therefore, establishing precise conditions for rTMS administration will be important for use in animal studies and clinical treatment as well as in vitro experiments. Stimulation of rTMS beyond the guaranteed safety conditions may be accompanied by pain and side effects.

Phosphorylation of components of intracellular signaling pathways contributes to cell survival by inhibiting signals associated with apoptosis and damage and, conversely, activating cytoprotective effects. Therefore, in an effort to elucidate the mechanism(s) underlying the therapeutic effects of rTMS, we investigated whether rTMS is involved in neuronal cell death, a primary cause of degenerative diseases. rTMS treatment exhibited neuroprotective effects in oxidative stress-induced cell death, as evidenced by high cell viability and suppressed neuronal cell death. Therefore, we suggest that rTMS can be applied as a therapeutic agent to relieve oxidative stress and treat neurodegenerative disorders.

ICV administration of STZ is accompanied by AD-like neuropathological and behavioral features, and is a model for exploring the etiology and developing therapies, particularly in sporadic AD research. There are few studies of the therapeutic efficacy of rTMS in animal models of sporadic AD. In this study, the efficacy of rTMS for AD was verified by focusing on the improvement in cognitive function and the cytoprotective effects as a treatment mechanism for rTMS. Our results showed that rTMS treatment in neurons mitigated STZ-induced apoptosis, synaptic loss, and hyperphosphorylation of tau. In addition, the results observed with the Y-maze test demonstrated that the cognitive decline observed in ICV-STZ rats was effectively improved when rTMS was applied. In the hippocampus, which is responsible for memory, rTMS treatment suppressed the histopathological features of AD induced by ICV-STZ administration, which is believed to be due to the cytoprotective effects of rTMS.

While rTMS has demonstrated potential as a therapeutic approach for the treatment of AD, it is crucial to acknowledge the remaining limitations. The efficacy of rTMS in treating AD is still under investigation, and although positive effects have been reported, the available evidence remains limited. Therefore, further research, including investigations into the mechanisms of action, is necessary. Additionally, the optimal parameters for rTMS therapy in AD have yet to be clearly defined, and standardized protocols have not been established. Moreover, there is inadequate research on the long-term effects and sustainability of rTMS in AD treatment. Another limitation is the variability of patient responses, as the effects of rTMS can vary based on individual factors such as disease stage, severity, and underlying brain pathology. Overall, rTMS holds potential as an effective treatment option for AD. However, to fully understand the potential benefits and limitations of rTMS in the context of AD treatment, further research is needed.

In summary, rTMS treatment activates the major intracellular signaling pathways STAT, MAPK, Akt/p70S6K, and CREB. These results suggest a mechanism through which rTMS exerts neuroprotective effects by increasing cell viability and anti-apoptotic effects. We systematically analyzed rTMS stimulation parameters and determined effective ranges and optimal conditions for efficient application of rTMS. These data will provide a useful basis for designing rTMS stimulation protocols for disease treatment. rTMS treatment improves memory and learning ability by increasing LTM-related gene expression, and alleviates cell damage and death caused by oxidative stress. Application of rTMS to AD-induced animal models improved memory deficits, inhibited cell death, and exhibited cytoprotective effects. Taken together, this study provides a novel mechanism for rTMS and may serve as a basis for explaining the efficacy of rTMS in the treatment of neurodegenerative diseases, including AD.

## Data Availability

The data and results generated in the current study are available from the corresponding author on reasonable request.
